# Serum immunoglobulin G predicts mortality and stratifies intravenous immunoglobulin benefit in sepsis patients

**DOI:** 10.1186/s40779-025-00657-5

**Published:** 2025-10-23

**Authors:** Yuan-Yuan Hu, Ming-Min Pang, Hao-Yu Wang, Wen-Xiong Li, Hao Wang

**Affiliations:** 1 Department of Critical Care Medicine, Qilu Hospital, Shandong University, Jinan, 250012 China; 2 Innovation Research Center for Sepsis and Multiple Organ Injury, Shandong University, Jinan, 250012 China; 3 Department of Critical Care Medicine, Beijing Chaoyang Hospital, Capital Medical University, Beijing, 100020 China

**Keywords:** Sepsis, Immunoglobulin G (IgG), Intravenous Immunoglobulin, Mortality

Dear Editor,

Sepsis is a major cause of mortality globally, driven by a dysregulated immune response that leads to multiorgan dysfunction [1]. The disease course typically progresses from an early hyperinflammatory state to prolonged immunosuppression, predisposing patients to secondary infections and excess death [2, 3]. Among the immunologic abnormalities, hypogammaglobulinemia, particularly low IgG, has been linked to poor outcomes but has not been used to guide adjunctive therapy. The efficacy of intravenous immunoglobulin (IVIg) in sepsis remains controversial: randomized trials have reported inconsistent results, and current guidelines advise against its routine use [4]. One major reason for these inconsistent findings is that most prior studies did not stratify patients by their baseline immune status, potentially masking benefits in selected subgroups [4–8].

We conducted a retrospective dual-cohort study using data from Qilu Hospital (2015 – 2025) and the Medical Information Mart for Intensive Care IV (MIMIC-IV) databases, applying Sepsis-3.0 criteria, to explore: 1) whether low serum IgG level is independently associated with 28-day mortality, and 2) whether IVIg treatment is associated with improved survival in patients with low IgG levels. Patients were stratified by serum IgG levels using a 670 mg/dl cut-off, with the primary outcome of 28-day mortality. Multivariable logistic regression, propensity score matching (PSM), restricted cubic splines, and receiver operating characteristic (ROC) curve analysis were employed. (Additional file 1: Methods).

In both the Qilu (*n* = 343) and MIMIC-IV (*n* = 1720) cohorts, patients with low serum IgG (< 670 mg/dl) had significantly higher 28-day mortality compared with those with higher IgG levels (Qilu: 53.3% vs. 29.3%; MIMIC-IV: 8.5% vs. 5.2%) (Additional file 1: Fig. S1 and Table S1). Across both cohorts, non-survivors had significantly lower IgG levels than survivors (Additional file 1: Table S2). Multivariable analyses confirmed that low IgG level was independently associated with 28-day mortality in Qilu (*OR* = 4.07, 95% CI 1.79–9.24) and MIMIC-IV (*OR* = 1.59, 95% CI 1.03–2.50) cohorts (Additional file 1: Tables S3 and S4). Sensitive analyses, including restricted cubic spline models, further supported the prognostic value of IgG (Additional file 1: Fig. S2 and Tables S5 and S6). In the MIMIC-IV subcohort with full immunological data (*n* = 322), IgG had the highest predictive value for 28-day mortality [area under the ROC curve (AUC) = 0.589], outperforming IgM and composite immunoglobulin parameters (Additional file 1: Table S7 and Fig. S3).

In both the Qilu and MIMIC-IV cohorts, there were no significant differences in 28-day mortality between IVIg recipients and non-recipients after PSM (*P* > 0.05) (Additional File 1: Tables S8 and S9). However, subgroup analysis demonstrated that IVIg therapy was associated with reduced 28-day mortality in patients with low IgG levels (*OR* = 0.19, 95% CI 0.06–0.56), but not in those with high IgG levels in Qilu cohort (Fig. 1). The similar differential effect of IVIg therapy on mortality between low and high IgG levels populations was also observed in MIMIC-IV cohort. Patients with high IgG levels even showed an increase in mortality rates when receiving IVIg therapy (Fig. 1). We further applied cohort-specific cut-off values to perform sensitive analyses. Notably, among patients with low IgG levels, IVIg treatment was associated with significantly reduced 28-day mortality in both cohorts even after PSM (Qilu: *OR* = 0.21, 95% CI 0.07–0.59; MIMIC-IV: *OR* = 0.29, 95% CI 0.10–0.75) (Additional file 1: Table S10). These results collectively suggest that hypogammaglobulinemia identifies a high-risk subgroup of sepsis patients and may indicate a potential survival benefit from IVIg therapy.

**Fig. 1:**
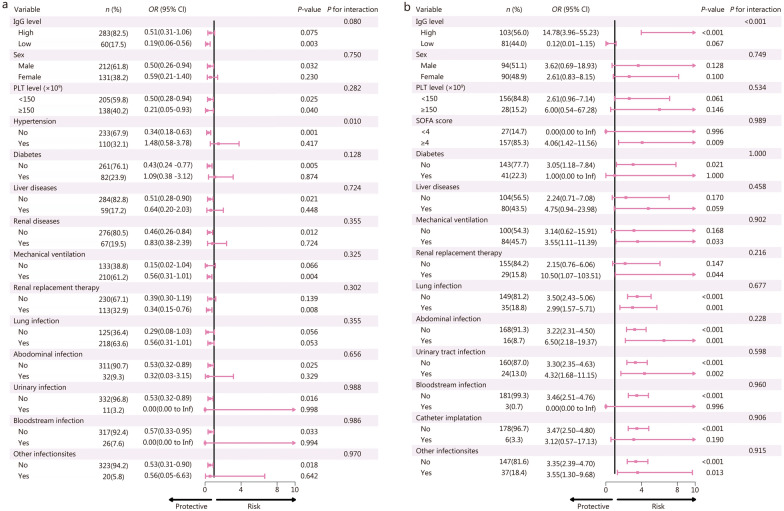
Subgroup analysis of IVIg efficacy on 28-day mortality in sepsis patients with different characteristics.** a** Qilu cohort. **b** MIMIC-IV cohort. CI confidence interval, OR odds ratio, IgG immunoglobulin G, MIMIC-IV Medical Information Mart for Intensive Care IV, PLT platelet, SOFA sequential organ failure assessment

Our study has several important implications. First, serum IgG is a simple, low-cost biomarker that can be quickly integrated into clinical workflows for early risk stratification. Second, unlike omics-based tools [9], IgG testing is feasible in real-time and resource-limited settings, offering a pragmatic tool for personalized immunotherapy. Third, we identified IgG deficiency as a potentially modifiable risk factor in sepsis, amenable to targeted IVIg therapy. Fourth, the differential effects of IVIg therapy observed between patients with low and high IgG levels suggest that IVIg treatment should be tailored according to the stratification of patients’ immune status. This work provides important clues to address the critical limitation of prior trials, the absence of immune stratification, which may have masked therapeutic effects in appropriate patients. Importantly, the subgroup analysis in low IgG level patients provides a preliminary clue regarding the potential efficacy of IVIg. Our findings suggest that future trials should incorporate baseline IgG testing to better define the population most likely to benefit from IVIg therapy.

Mechanistically, hypogammaglobulinemia in sepsis may arise not from impaired immunoglobulin synthesis but from immune-mediated consumption and redistribution secondary to vascular leakage and endothelial dysfunction. In this context, IVIg supplementation may restore humoral immunity by replenishing depleted IgG pools, thereby rebalancing immune homeostasis and improving outcomes.

Nevertheless, this study has several limitations. First, the retrospective design of this study limits the ability to draw causal inferences. Second, IVIg exposure was recorded as binary, without details on dosing, formulation, timing, or clinical indications—critical factors for efficacy assessment. Third, despite rigorous adjustment via multivariable regression and PSM, residual confounding cannot be excluded. Finally, the absence of longitudinal IgG measurements limits the understanding of dynamic immune changes during sepsis, and incomplete immunophenotyping (e.g., T/B cell status, cytokine profiles) limits the ability to further refine patient stratification. Furthermore, the discrepancies observed between the two cohorts in subgroup analyses likely reflect substantial heterogeneity. In China, IgG testing and IVIg treatment are not routine and are mainly used in the most critically ill ICU patients, contributing to the greater severity seen in the Qilu cohort. Considering this heterogeneity, limited sample sizes, and restricted external validity, the observed reduction in 28-day mortality with IVIg treatment in patients with low IgG levels should be regarded as a preliminary result and interpreted with caution. Further validation in large multicenter cohorts is needed, and if feasible, a prospective interventional trial should be conducted to confirm the causal relationship.

In conclusion, this study highlights the prognostic and therapeutic importance of IgG in sepsis. We propose that IVIg may benefit a specific subgroup of patients, those with low IgG levels, who are currently overlooked in clinical guidelines. These findings support the rationale for using IgG to guide patient stratification in a randomized controlled trial. We propose a stepwise approach to precision immunotherapy, beginning with readily accessible, low-cost clinical or laboratory markers, such as serum IgG levels, for early risk stratification. High-risk patients, such as sepsis patients with low IgG levels, can then receive targeted interventions, including IVIg therapy, followed by more advanced evaluations (e.g., immunologic or molecular diagnostics) as clinically indicated. This tiered strategy can be integrated into existing clinical workflows, enabling timely identification of high-risk patients, efficient triage, and optimized resource utilization.

## Supplementary Information

**Additional file 1.** Methods. **Fig. S1** Participant recruitment flowchart. **Fig. S2** Restricted cubic spline (RCS) regression for baseline serum IgG levels in relation to 28-day mortality. **Fig. S3** ROC curve comparison of immunological markers for predicting 28-day mortality in the MIMICIV cohort. **Table S1** Baseline characteristics of the Qilu and MIMIC-IV cohorts of sepsis patients by IgG levels (cut-off 670 mg/dl). **Table S2** Univariate analysis of factors associated with 28-day mortality in sepsis patients. **Table S3** Multivariable logistic regression models evaluating the association between low serum IgG levels and 28-day mortality in the Qilu cohort. **Table S4** Multivariable logistic regression models evaluating the association between low serum IgG levels and 28-day mortality in the MIMIC-IV cohort. **Table S5** Multivariable logistic regression models evaluating the association between low serum IgG levels ( < 656 mg/dl) and 28-day mortality in the Qilu cohort. **Table S6** Multivariable logistic regression models evaluating the association between low serum IgG levels ( < 694 mg/dl) and 28-day mortality in the MIMIC-IV cohort. **Table S7** Comparison of area under the ROC curve (AUC) values for predicting outcome using IgG and other immunological parameters. **Table S8** Baseline characteristics of sepsis patients with and without IVIg treatment in the Qilu cohort before and after PSM. **Table S9** Baseline characteristics of sepsis patients with and without IVIg treatment in the MIMIC-IV cohort before and after PSM. **Table S10** Association of IVIg treatment with 28-day mortality after PSM in low IgG patients in the Qilu and MIMIC-IV cohorts [*n* (%)].

## Data Availability

The datasets presented in this article are not publicly available, as they were obtained from the MIMIC-IV clinical database. Access to these datasets can be requested through PhysioNet (www.physionet.org/). The internal cohort datasets used and analyzed during the current study are available from the corresponding author upon reasonable request.

## Abbreviations

AUC: Area under the ROC curve
CI: Confidence interval
IgG: Immunoglobulin G
IVIg: Intravenous immunoglobulin
MIMIC-IV: Medical Information Mart for Intensive Care IV
OR: Odds ratio
PSM: Propensity score matching
ROC: Receiver operating characteristic
