# Supplementary material for: Serum immunoglobulin G predicts mortality and stratifies intravenous immunoglobulin benefit in sepsis patients
Source: Mil Med Res. 2025 Oct 23;12:70. doi: 10.1186/s40779-025-00657-5 (PMC12551274; doi:10.1186/s40779-025-00657-5)
Supplement: Supplementary file 1 — Additional file 1. Methods. Fig. S1 Participant recruitment flowchart. Fig. S2 Restricted cubic spline (RCS) regression for baseline serum IgG levels in relation to 28-day mortality. Fig. S3 ROC curve comparison of immunological markers for predicting 28-day mortality in the MIMICIV cohort. Table S1 Baseline characteristics of the Qilu and MIMIC-IV cohorts of sepsis patients by IgG levels (cut-off 670 mg/dl). Table S2 Univariate analysis of factors associated with 28-day mortality in sepsis patients. Table S3 Multivariable logistic regression models evaluating the association between low serum IgG levels and 28-day mortality in the Qilu cohort. Table S4 Multivariable logistic regression models evaluating the association between low serum IgG levels and 28-day mortality in the MIMIC-IV cohort. Table S5 Multivariable logistic regression models evaluating the association between low serum IgG levels ( < 656 mg/dl) and 28-day mortality in the Qilu cohort. Table S6 Multivariable logistic regression models evaluating the association between low serum IgG levels ( < 694 mg/dl) and 28-day mortality in the MIMIC-IV cohort. Table S7 Comparison of area under the ROC curve (AUC) values for predicting outcome using IgG and other immunological parameters. Table S8 Baseline characteristics of sepsis patients with and without IVIg treatment in the Qilu cohort before and after PSM. Table S9 Baseline characteristics of sepsis patients with and without IVIg treatment in the MIMIC-IV cohort before and after PSM. Table S10 Association of IVIg treatment with 28-day mortality after PSM in low IgG patients in the Qilu and MIMIC-IV cohorts [n (%)]. [file 40779_2025_657_MOESM1_ESM.pdf]

Methods

Study design and population

This was a retrospective dual-cohort study utilizing data from two independent datasets. The internal cohort was collected from Qilu Hospital of Shandong University between May 2015 and May 2025, while the validation cohort was included from the Medical Information Mart for Intensive Care IV (MIMIC-IV) database [1, 2]. MIMIC-IV is a longitudinal, single-center database including 364,627 unique individuals, 546,028 hospitalizations, and 94,458 unique intensive care unit (ICU) stays. Ming-Min Pang (Record ID 11057215) is certified to get access to the database and is responsible for data extraction. The databases contain demographic data, lab results, nursing notes, intravenous medications, fluid balance, and other clinical variables, with ICD-9 and ICD-10 codes for specific diseases. Sepsis patients (≥ 18 years old) were identified based on the sepsis 3.0 diagnostic criteria, including infection and a sequential organ failure assessment (SOFA) score ≥ 2 [3]. Exclusion criteria included ICU stays of less than 48 h, multiple ICU admissions (only the first admission was included), intravenous immunoglobulin (IVIg) not used for treating infection, and missing data exceeding 20% for IVIg usage or other variables (**Fig. S1**).

Selection of the IgG threshold

Dose-response analyses were performed independently in the Qilu and MIMIC-IV cohorts to identify optimal immunoglobulin G (IgG) thresholds for mortality prediction. The best-performing threshold identified in the Qilu cohort was 694 mg/dl, while in the MIMIC-IV cohort it was 656 mg/dl (**Fig. S2**). Consistent with established literature [4, 5] identifying a specific optimal IgG level, and considering that this literature-based value represented the midpoint between the cohort-specific optima derived from the analyses, we adopted the literature-based IgG threshold of 670 mg/dl for subsequent analyses in this study.

Use of IVIg and clinical outcomes

IVIg therapy was defined by the administration of drugs including Gammagard, Privigen, Flebogamma, Gamunex, Hizentra (subcutaneous Ig), Octagam, Intratect, Kiovig, Panzyga, Venoglobulin, Cuvitru (subcutaneous Ig), and Gammaplex. Medication prescriptions were recorded through the provider order interface. Only data from each patient's first ICU admission were included. Sepsis patients were stratified into high and low IgG groups based on IgG concentrations measured

after sepsis diagnosis, using a 670 mg/dl cut-off [4, 5]. The primary outcome was 28-day all-cause mortality, defined as death within 28 d of ICU admission.

Covariates

Demographic and admission information, including age, gender, race, admission day and time, and severity at admission [simplified acute physiology score (SAPS), acute physiology and chronic health evaluation II (APACHE II), and SOFA score] were recorded. Comorbidities, such as congestive heart failure, chronic obstructive pulmonary disease, diabetes, renal failure, liver disease, and solid tumors, were identified using ICD codes. Interventions such as mechanical ventilation (MV) and continuous renal replacement therapy (CRRT) were also included.

Statistical analysis

Continuous variables were presented as mean ± standard deviation or median (interquartile range) [median (IQR)], depending on the distribution, while categorical variables were expressed as proportions. The Kolmogorov-Smirnov test was employed to evaluate normality. Comparisons between continuous variables were made using the Mann-Whitney *U* test or the Kruskal-Wallis test for non-normally distributed data. Binary logistic regression was performed to assess factors influencing all-cause 28-day mortality risk. Multivariable logistic models were used to calculate the odds ratio (*OR*) and 95% confidence interval (CI) for the association between IgG and sepsis mortality, adjusting for confounding variables, including risk factors and baseline characteristics. Clinically relevant and prognosis-associated variables were incorporated into the models. In Qilu cohort, Model 1: IgG + age + gender, Model 2: Model 1 + APACHE II +SOFA + MV + CRRT, and Model 3: Model 2 + comorbidities. In MIMIC cohort, Model 1: IgG + age + gender + race, Model 2: Model 1 + SOFA + MV+ CRRT, Model 3: Model 2 + comorbidities. Propensity score matching (PSM) was used to balance covariates between the high IgG group and the low IgG group in the MIMIC cohort.

To characterize the dose-response relationship between IgG level and 28-day mortality, we employed restricted cubic splines (RCS) with 4 knots positioned at the 5th, 35th, 65th, and 95th percentiles within fully-adjusted models. Nonlinearity was formally assessed using the likelihood ratio test. This analytical approach was implemented independently in both cohorts. To compare the predictive value of IgG with other immunological indicators, we performed receiver operating characteristic (ROC) curve analysis in a subcohort with complete immunological data (n = 322) in the MIMIC-IV cohort. We performed ROC curve analyses comparing IgG against IgA, IgM, CD3,

CD4, CD8, and the CD4/CD8 ratio. Furthermore, we constructed composite immunoglobulin parameters: two-class sums [IgG + IgM (denoted IgGM), IgG + IgA (denoted IgAG), IgA + IgM (denoted IgAM)] and a three-class sum [IgG + IgA + IgM (denoted IgAGM)]. To ensure dimensional homogeneity and comparability across these composite scores, all contributing Ig values (IgG, IgA, and IgM) were *Z*-score standardized before summation. To assess the significance of performance differences relative to the base IgG concentration, we employed DeLong's test for paired ROC curves. *P*-values were adjusted for multiple comparisons using the Bonferroni method.

Subgroup analyses were performed separately in both the Qilu and MIMIC-IV cohorts to examine the heterogeneity of the IVIg treatment effect on 28-day mortality across prespecified clinical strata.

We further evaluated the efficacy of IVIg in the subgroup of patients with low serum IgG. Comparisons of 28-day mortality between IVIg-treated and untreated patients were performed using the same 1:1 PSM strategy in both cohorts. The propensity score was calculated using logistic regression, where the dependent variable was IVIg treatment (yes/no), and the independent variables included demographic characteristics, clinical variables, and comorbidities relevant to patients. Matching was performed using the nearest-neighbor method without replacement, with a caliper width of 0.2 standard deviations of the logit of the propensity score. A two-sided $P < 0.05$ was considered statistically significant. All analyses were performed using R software (version 4.3.3).

References

1. Johnson AEW, Bulgarelli L, Shen L, Gayles A, Shammout A, Horng S, *et al*. MIMIC-IV, a freely accessible electronic health record dataset. Sci Data. 2023;10(1):1.
2. Johnson A, Bulgarelli L, Pollard T, Gow B, Moody B, Horng S, *et al*. MIMIC-IV (version 3.1). PhysioNet. RRID:SCR_007345. https://doi.org/10.13026/kpb9-mt58.
3. Singer M, Deutschman CS, Seymour CW, Shankar-Hari M, Annane D, Bauer M, *et al*. The third international consensus definitions for sepsis and septic shock (Sepsis-3). JAMA. 2016;315(8):801-10.
4. Akatsuka M, Masuda Y, Tatsumi H, Sonoda T. Efficacy of intravenous immunoglobulin therapy for patients with sepsis and low immunoglobulin G levels: a single-center retrospective study. Clin Ther. 2022;44(2):295-303.
5. Akatsuka M, Tatsumi H, Sonoda T, Masuda Y. Low immunoglobulin G level is associated with poor outcomes in patients with sepsis and septic shock. J Microbiol Immunol Infect. 2021;54(4):728-32.

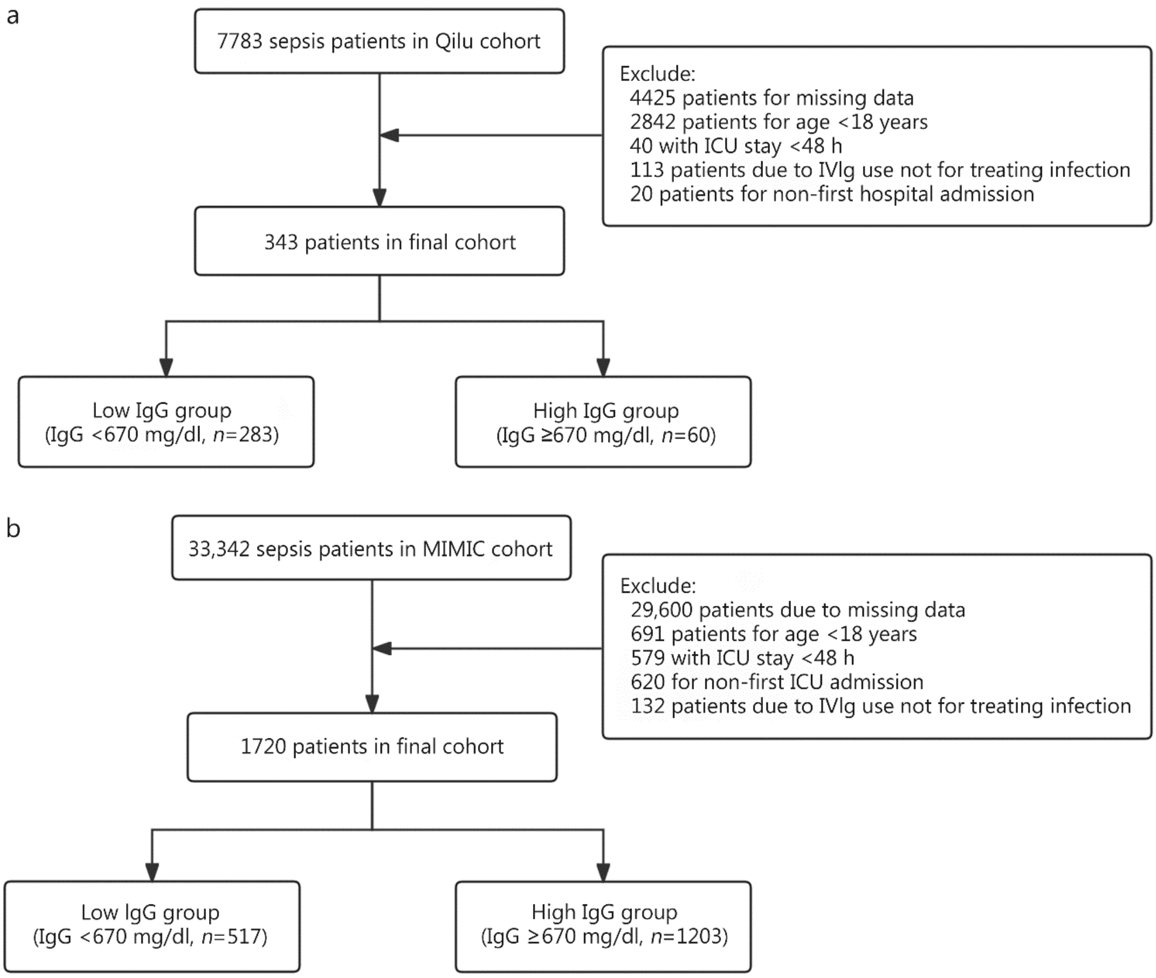

Fig. S1 Participant recruitment flowchart. **a** Qilu cohort: internal data from Qilu Hospital of Shandong University. **b** MIMIC-IV cohort. ICU intensive care unit, IgG immunoglobulin G, IVIg intravenous immunoglobulin, MIMIC-IV Medical Information Mart for Intensive Care IV

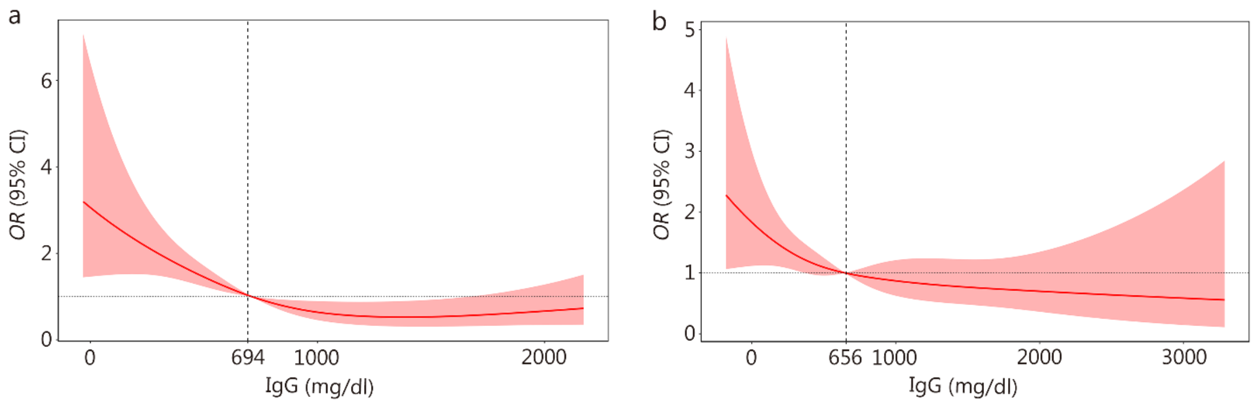

Fig. S2 Restricted cubic spline (RCS) regression for baseline serum IgG levels in relation to 28-day mortality. **a** Qilu cohort: the optimal cut-off value derived was 694 mg/dl. *P*-overall < 0.001, *P*-nonlinear < 0.001. **b** MIMIC-IV cohort: the optimal cut-off value derived was 656 mg/dl. *P*-overall < 0.001, *P*-nonlinear = 0.563. CI confidence interval, OR odds ratio, IgG immunoglobulin G, MIMIC-IV Medical Information Mart for Intensive Care IV

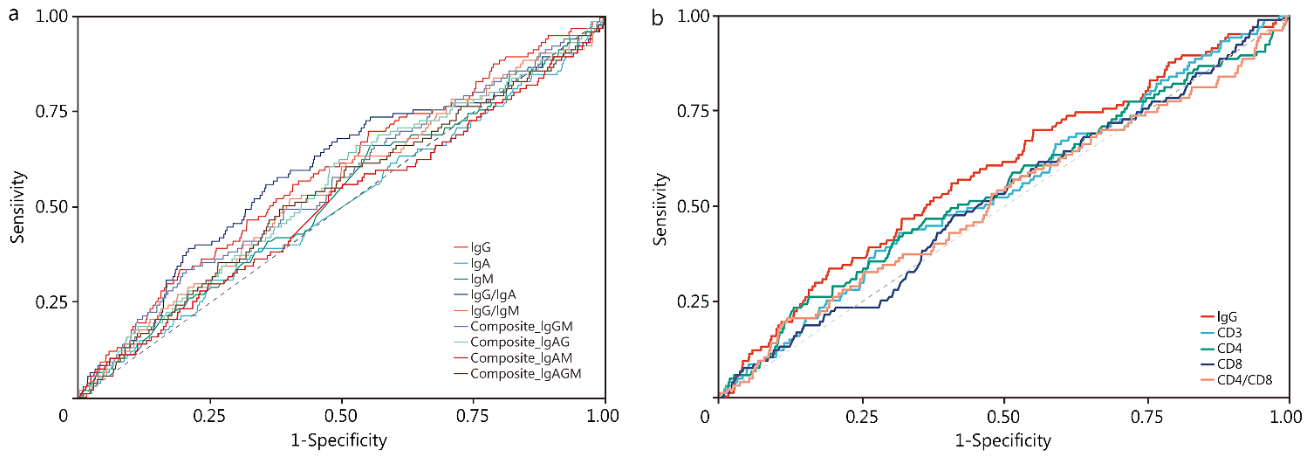

Fig. S3 ROC curve comparison of immunological markers for predicting 28-day mortality in the MIMIC-IV cohort. **a** Comparison of individual Ig (IgG, IgA, and IgM), Ig ratios (IgG/IgA, IgG/IgM), and composite Ig scores (Composite_IgAG, Composite_IgGM, Composite_IgAM, and Composite_IgAGM). **b** Comparison of IgG and lymphocyte subset markers (CD3, CD4, CD8, and CD4/CD8 ratio). CD3 cluster of differentiation 3, CD4 cluster of differentiation 4, CD8 cluster of differentiation 8, CD4/CD8 ratio CD4 to CD8 T cell ratio, IgG immunoglobulin G, IgA immunoglobulin A, IgM immunoglobulin M, IgGM immunoglobulin G and M, IgAG immunoglobulin A and G, IgAM immunoglobulin A and M, IgAGM immunoglobulin A, G, and M, IgG/IgA IgG to IgA ratio, IgG/IgM IgG to IgM ratio, MIMIC-IV Medical Information Mart for Intensive Care IV, ROC receiver operating characteristic

Table S1 Baseline characteristics of the Qilu and MIMIC-IV cohorts of sepsis patients by IgG levels (cut-off 670 mg/dl)

Variables	Qilu cohort				MIMIC-IV cohort			
	**Total (*n* = 343)**	**Low IgG (*n* = 283)**	**High IgG (*n* = 60)**	***P*-value**	**Total (*n* = 1720)**	**Low IgG (*n* = 517)**	**High IgG (*n* = 1203)**	***P*-value**
Male [*n* (%)]	212 (61.8)	36 (60.0)	176 (62.2)	0.864	958 (55.7)	289 (55.9)	669 (55.6)	0.954
Age [years, median (IQR)]	62.0 (51.0 – 72.0)	62.0 (54.0 –71.2)	62.0 (50.0 – 73.0)	0.866	63.1 (52.8 – 71.8)	64.7 (55.8 – 73.7)	61.9 (51.5 – 70.9)	< 0.001
CCI [median (IQR)]	-	-	-	-	7.0 (5.0 – 9.0)	8.0 (6.0 – 9.0)	7.0 (5.0 – 9.0)	0.098
Platelet [$\times 10^9$/L, median (IQR)]	109.0 (45.5 – 216.0)	72.0 (34.2 – 138.0)	126.0 (51.5 – 243.0)	0.001	83.0 (34.0 – 143.0)	92.0 (43.5 – 151.0)	58.0 (15.0 – 126.0)	< 0.001
IgG level [mg/dl, median (IQR)]	1090.0 (777.0 – 1510.0)	480.0 (389.0 – 553.0)	1240.0 (977.0 – 1590.0)	< 0.001	938.0 (593.0 – 1359.0)	435.0 (300.0 – 549.0)	1148.0 (904.0 – 1586.0)	< 0.001
Comorbidities								
Hypertension [*n* (%)]	110 (32.1)	23 (38.3)	87 (30.7)	0.321	-	-	-	-
Congestive heart failure [*n* (%)]	-	-	-	-	762 (44.3)	236 (45.6)	526 (43.7)	0.494
COPD [*n* (%)]	-	-	-	-	746 (43.4)	249 (48.2)	497 (41.3)	0.010
Diabetes [*n* (%)]	82 (23.9)	19 (31.7)	63 (22.3)	0.166	640 (37.2)	176 (34.0)	464 (38.6)	0.084
Renal failure [*n* (%)]	67 (19.5)	16 (26.7)	51 (18.0)	0.175	778 (45.2)	237 (45.8)	541 (45.0)	0.780
Liver disease [*n* (%)]	59 (17.2)	11 (18.3)	48 (17.0)	0.946	712 (41.4)	153 (29.6)	559 (46.5)	< 0.001

Variables	Qilu cohort				MIMIC-IV cohort			
	**Total (*n* = 343)**	**Low IgG (*n* = 283)**	**High IgG (*n* = 60)**	***P*-value**	**Total (*n* = 1720)**	**Low IgG (*n* = 517)**	**High IgG (*n* = 1203)**	***P*-value**
Solid tumor [*n* (%)]	-	-	-	-	115 (6.69)	37 (7.2)	78 (6.48)	0.684
Severity of illness								
MV [*n* (%)]	210 (61.2)	45 (75.0)	165 (58.3)	0.024	693 (40.3)	184 (35.6)	509 (42.3)	0.011
CRRT [*n* (%)]	113 (32.9)	29 (48.3)	84 (29.7)	0.008	133 (7.7)	36 (7.0)	97 (8.1)	0.494
28-day mortality [*n* (%)]	115 (33.5)	32 (53.3)	83 (29.3)	0.001	107 (6.2)	44 (8.5)	63 (5.2)	0.014
Length of stay in hospital [d, median (IQR)]	18.0 (11.0 – 29.0)	18.0 (11.0 – 29.0)	18.5 (9.8 – 27.2)	0.877	11.5 (6.3 – 23.6)	14.5 (7.3 – 28.0)	10.5 (6.0 – 21.1)	< 0.001
APS III [median (IQR)]	-	-	-	-	62.0 (46.0 – 83.0)	63.0 (47.0 – 85.0)	61.0 (46.0 – 83.0)	0.298
SOFA score [median (IQR)]	9.0 (6.75 – 12.0)	9.0 (7.0 – 12.0)	9.0 (6.0 – 12.0)	0.941	6.0 (4.0 – 9.0)	6.0 (4.0 – 9.0)	6.0 (4.0 – 9.0)	0.275
APACHE II [median (IQR)]	25.0 (19.0 – 29.0)	25.0 (20.0 – 30.0)	25.0 (19.0 – 28.2)	0.339	-	-	-	-
IVIg treatment [*n* (%)]	107 (31.2)	79 (27.9)	28 (46.7)	0.007	92 (5.35)	40 (3.33)	52 (10.1)	< 0.001

Data were presented as *n* (%) for categorical variables and median (IQR) for continuous variables. *APACHE II* acute physiology and chronic health evaluation II, *APS III* acute physiology score III, *CCI* Charlson comorbidity index, *COPD* chronic obstructive pulmonary disease, *CRRT* continuous renal replacement therapy, *IgG* immunoglobulin G, *IQR* interquartile range*, IVIg* intravenous immunoglobulin, *MIMIC-IV* Medical Information Mart for Intensive Care IV, *MV* mechanical ventilation, *SOFA* sequential organ failure assessment

Table S2 Univariate analysis of factors associated with 28-day mortality in sepsis patients

Variables	Qilu cohort				MIMIC-IV cohort			
	**Total (*n* = 343)**	**Survivors (*n* = 228)**	**Non-survivors (*n* = 115)**	***P*-value**	**Total (*n* = 1720)**	**Survivors (*n* = 1617)**	**Non-survivors (*n* = 107)**	***P*-value**
Male [*n* (%)]	212 (61.8)	136 (59.6)	76 (66.1)	0.298	958 (55.7)	903 (56.0)	55 (51.4)	0.410
Age [years, median (IQR)]	62.0 (51.0 – 72.0)	61.0 (49.0 – 71.0)	65.0 (53.5 – 74.0)	0.038	63.1 (52.8 – 71.8)	62.8 (52.5 – 71.5)	67.0 (58.3 – 77.2)	0.001
Platelet [$\times 10^9$/L, median (IQR)]	109.0 (45.5 – 216.0)	149.0 (66.0 – 272.0)	64.0 (31.5 – 131.0)	< 0.001	83.0 (34.0 – 143.0)	86.0 (37.0 – 144.0)	39.0 (13.0 – 82.5)	< 0.001
IgG level [mg/dl, median (IQR)]	1090.0 (777.0 – 1510.0)	1170.0 (910.0 – 1560.0)	963 .0 (630.0 – 1380.0)	< 0.001	938.0 (593.0 – 1359.0)	941.0 (605.0 – 1366.0)	747.0 (442.0 – 1155.0)	0.002
MV [*n* (%)]	210 (61.2)	119 (52.2)	91 (79.1)	< 0.001	693 (40.3)	636 (39.4)	57 (53.3)	0.006
CRRT [*n* (%)]	113 (32.9)	64 (28.1)	49 (42.6)	0.001	133 (7.7)	113 (7.0)	20 (18.7)	< 0.001
Length of stay in hospital [d, median (IQR)]	18.0 (11.0 – 29.0)	23.0 (13.0 – 35.2)	12.0 (6.0 – 19.0)	< 0.001	11.5 (6.3 – 23.6)	10.9 (6.0 – 22.0)	22.8 (13.7 – 37.5)	< 0.001
APS III [median (IQR)]	-	-	-	-	62.0 (46.0 – 83.0)	60.0 (45.0 – 81.0)	85.0 (67.5 – 109.0)	< 0.001
SOFA score [median (IQR)]	9.0 (6.8 – 12.0)	9.0 (6.0 – 11.0)	10.0 (7.5 – 14.0)	0.011	6.0 (4.0 – 9.0)	6.0 (4.0 – 9.0)	9.0 (6.0 – 14.0)	< 0.001
APACHE II [median (IQR)]	25.0 (19.0 – 29.0)	24.0 (18.0 – 28.0)	26.0 (21.8 – 30.2)	0.016	-	-	-	-

Variables	Qilu cohort				MIMIC-IV cohort			
	**Total (*n* = 343)**	**Survivors (*n* = 228)**	**Non-survivors (*n* = 115)**	***P*-value**	**Total (*n* = 1720)**	**Survivors (*n* = 1617)**	**Non-survivors (*n* = 107)**	***P*-value**
Hypertension [*n* (%)]	110 (32.1)	80 (35.1)	30 (26.1)	0.118	-	-	-	-
Congestive heart failure [*n* (%)]	-	-	-	-	762 (44.3)	717 (44.5)	45 (42.1)	0.702
COPD [*n* (%)]	-	-	-	-	746 (43.4)	711 (44.1)	35 (32.7)	0.028
Diabetes [*n* (%)]	82 (23.9)	57 (25.0)	25 (21.7)	0.593	640 (37.2)	618 (38.3)	22 (20.6)	< 0.001
Renal failure [*n* (%)]	67 (19.5)	38 (16.7)	29 (25.2)	0.082	778 (45.2)	747 (46.3)	31 (29.0)	0.001
Liver disease [*n* (%)]	59 (17.2)	35 (15.4)	24 (20.9)	0.260	712 (41.4)	671 (41.6)	41 (38.3)	0.571
Solid tumor [*n* (%)]	-	-	-	-	115 (6.7)	106 (6.6)	9 (8.4)	0.591

Data were presented as *n* (%) for categorical variables and median (IQR) for continuous variables. *APACHE II* acute physiology and chronic health evaluation II, *APS III* acute physiology score III, *CCI* Charlson comorbidity index, *COPD* chronic obstructive pulmonary disease, *CRRT* continuous renal replacement therapy, *IgG* immunoglobulin G, *IQR* interquartile range*, IVIg* intravenous immunoglobulin, *MIMIC-IV* Medical Information Mart for Intensive Care IV, *MV* mechanical ventilation, *SOFA* sequential organ failure assessment

Table S3 Multivariable logistic regression models evaluating the association between low serum IgG levels and 28-day mortality in the Qilu cohort

**Model**	***OR* (95% CI)**	***P*-value**
Model 1	2.76 (1.56 – 4.89)	0.007
Model 2	3.93 (1.76 – 8.78)	< 0.001
Model 3	4.07 (1.79 – 9.24)	< 0.001

Variables were adjusted: Model 1: IgG + age + sex; Model 2: Model 1 + APACHE II + SOFA + MV + CRRT; Model 3: Model 2 + comorbidities. *APACHE II* acute physiology and chronic health evaluation II, *CI* confidence interval, *CRRT* continuous renal replacement therapy, *IgG* immunoglobulin G, *MV* mechanical ventilation, *OR* odds ratio, *SOFA* sequential organ failure assessment

Table S4 Multivariable logistic regression models evaluating the association between low serum IgG levels and 28-day mortality in the MIMIC-IV cohort

**Model**	***OR* (95% CI)**	***P*-value**	***OR* (95% CI) after PSM**	***P*-value**
Model 1	1.69 (1.12 – 2.50)	< 0.001	1.64 (1.12 – 2.50)	< 0.001
Model 2	1.56 (1.03 – 2.32)	< 0.001	1.56 (1.03 – 2.42)	< 0.001
Model 3	1.59 (1.03 – 2.50)	< 0.001	1.59 (1.03 – 2.50)	< 0.001

Variables were adjusted: Model 1: IgG + age + sex + race; Model 2: Model 1 + SOFA + MV + CRRT; Model 3: Model 2 + comorbidities. *CI* confidence interval, *CRRT* continuous renal replacement therapy, *IgG* immunoglobulin G, *MIMIC-IV* Medical Information Mart for Intensive Care IV, *MV* mechanical ventilation, *OR* odds ratio, *PSM* propensity score matching, *SOFA* sequential organ failure assessment

Table S5 Multivariable logistic regression models evaluating the association between low serum IgG levels (< 656 mg/dl) and 28-day mortality in the Qilu cohort

**Model**	***OR* (95%CI)**	***P*-value**
Model 1	1.74 (1.17 – 2.58)	< 0.001
Model 2	1.64 (1.10 – 2.44)	< 0.001
Model 3	1.73 (1.14 – 2.62)	< 0.001

Variables were adjusted: Model 1: IgG + age + sex; Model 2: Model 1 + APACHE II +SOFA + MV + CRRT; Model 3: Model 2 + comorbidities. *APACHE II* acute physiology and chronic health evaluation II, *CI* confidence interval, *CRRT* continuous renal replacement therapy, *IgG* immunoglobulin G, *MV* mechanical ventilation, *OR* odds ratio, *SOFA* sequential organ failure assessment

Table S6 Multivariable logistic regression models evaluating the association between low serum IgG levels (< 694 mg/dl) and 28-day mortality in the MIMIC-IV cohort

**Model**	***OR* (95%CI)**	***P*-value**
Model 1	3.45 (1.99 – 5.97)	< 0.001
Model 2	5.37 (2.47 – 11.67)	< 0.001
Model 3	5.76 (2.60 – 12.78)	< 0.001

Variables were adjusted: Model 1: IgG + age + sex + race; Model 2: Model 1 + SOFA + MV + CRRT; Model 3: Model 2 + comorbidities. *CI* confidence interval, *CRRT* continuous renal replacement therapy, *IgG* immunoglobulin G, *MIMIC-IV* Medical Information Mart for Intensive Care IV, *MV* mechanical ventilation, *OR* odds ratio, *SOFA* sequential organ failure assessment

Table S7 Comparison of area under the ROC curve (AUC) values for predicting outcome using IgG and other immunological parameters

**Variables**	**AUC**	**Difference**	***P*-value**	**Significance**
IgG	0.589	Ref.	-	-
IgA	0.501	0.089	0.082	ns
IgM	0.526	0.116	0.017	$P < 0.05$
IgG/IgA	0.593	0.004	0.929	ns
IgG/IgM	0.550	0.039	0.280	ns
Composite_IgGM	0.561	0.028	0.099	ns
Composite_IgAG	0.552	0.038	0.009	$P < 0.01$
Composite_IgAM	0.506	0.083	0.009	$P < 0.01$
Composite_IgAGM	0.537	0.053	0.013	$P < 0.05$
CD3	0.543	0.047	0.242	ns
CD4	0.545	0.045	0.269	ns
CD8	0.518	0.072	0.088	ns
CD4/CD8	0.516	0.073	0.064	ns

CD3 cluster of differentiation 3, *CD4* cluster of differentiation 4, *CD8* cluster of differentiation 8, *CD4/CD8* CD4 to CD8 T cell ratio, *IgG* immunoglobulin G, *IgA* immunoglobulin A, *IgM* immunoglobulin M, *IgGM* immunoglobulin G and M, *IgAG* immunoglobulin A and G, *IgAM* immunoglobulin A and M, *IgAGM* immunoglobulin A, G, and M, *IgG/IgA* IgG to IgA ratio, *IgG/IgM* IgG to IgM ratio, *ROC* receiver operating characteristic, *ns* non-significant, "-" indicated meaningless or absent

Table S8 Baseline characteristics of sepsis patients with and without IVIg treatment in the Qilu cohort before and after PSM

Variables	Qilu cohort				Qilu cohort after PSM			
	**Total (*n* = 343)**	**Non-IVIg (*n* = 236)**	**IVIg (*n* = 107)**	***P*-value**	**Total (*n* = 214)**	**Non-IVIg (*n* = 107)**	**IVIg (*n* = 107)**	***P*-value**
IgG level [mg/dl, median (IQR)]	1090.0 (777.0 – 1510.0)	1110.0 (842.0 – 1520.0)	1050.0 (646.0 – 1490.0)	0.203	1080.0 (759.0 – 1490.0)	1100.0 (874.0 – 1490.0)	1050.0 (646.0 – 1490.0)	0.280
28-day mortality [*n* (%)]	115 (33.5)	89 (37.7)	26 (24.3)	0.021	53 (24.8)	27 (25.2)	26 (24.3)	> 0.999
APACHE II [median (IQR)]	25.0 (19.0 – 29.0)	24.0 (19.0 – 29.0)	26.0 (20.8 – 30.0)	0.371	13.0 (5.0 – 25.0)	15.0 (5.0 – 25.0)	5.0 (5.0 – 26.0)	0.369
SOFA score [median (IQR)]	9.0 (6.8 – 12.0)	9.0 (7.0 – 12.0)	10.0 (6.0 – 12.0)	0.991	4.50 (1.0 – 9.0)	6.0 (1.0 – 9.0)	1.00 (1.0 – 9.5)	0.232
MV [*n* (%)]	210 (61.2)	129 (54.7)	81 (75.7)	< 0.001	156 (72.9)	75 (70.1)	81 (75.7)	0.442
CRRT [*n* (%)]	113 (32.9)	67 (28.4)	46 (43.0)	0.011	85 (39.7)	39 (36.4)	46 (43.0)	0.402
Male [*n* (%)]	212 (61.8)	145 (61.4)	67 (62.6)	0.930	130 (60.7)	63 (58.9)	67 (62.6)	0.675
Age [years, median (IQR)]	62.0 (51.0 – 72.0)	64.0 (55.0 – 73.0)	56.0 (40.0 – 70.0)	0.001	58.0 (43.0 – 69.0)	60.0 (49.0 – 68.0)	56.0 (40.0 – 70.0)	0.338
Platelet [$\times 10^9$/L, median (IQR)]	109.0 (45.5 – 216.0)	127.0 (60.0 – 226.0)	72.0 (34.5 – 176.0)	0.005	87.0 (39.0 – 194.0)	107.0 (44.0 – 198.0)	72.0 (34.5 – 176.0)	0.262
Diabetes [*n* (%)]	82 (23.9)	60 (25.4)	22 (20.6)	0.400	45 (21.0)	23 (21.5)	22 (20.6)	> 0.999
Hypertension [*n* (%)]	110 (32.1)	83 (35.2)	27 (25.2)	0.089	65 (30.4)	38 (35.5)	27 (25.2)	0.137

Variables	Qilu cohort				Qilu cohort after PSM			
	**Total (*n* = 343)**	**Non-IVIg (*n* = 236)**	**IVIg (*n* = 107)**	***P*-value**	**Total (*n* = 214)**	**Non-IVIg (*n* = 107)**	**IVIg (*n* = 107)**	***P*-value**
Liver disease [*n* (%)]	59 (17.2)	41 (17.4)	18 (16.8)	> 0.999	40 (18.7)	22 (20.6)	18 (16.8)	0.599
Renal failure [*n* (%)]	67 (19.5)	47 (19.9)	20 (18.7)	0.906	37 (17.3)	17 (15.9)	20 (18.7)	0.718

Data were presented as *n* (%) for categorical variables and median (IQR) for continuous variables. *APACHE II* acute physiology and chronic health evaluation II, *CRRT* continuous renal replacement therapy, *IgG* immunoglobulin G, *IQR* interquartile range*, IVIg* intravenous immunoglobulin, *MV* mechanical ventilation, *PSM* propensity score matching, *SOFA* sequential organ failure assessment

Table S9 Baseline characteristics of sepsis patients with and without IVIg treatment in the MIMIC-IV cohort before and after PSM

Variables	MIMIC-IV cohort				MIMIC-IV cohort after PSM			
	**Total (*n* = 1720)**	**Non-IVIg (*n* = 1628)**	**IVIg (*n* = 92)**	***P*-value**	**Total (*n* = 184)**	**Non-IVIg (*n* = 92)**	**IVIg (*n* = 92)**	***P*-value**
IgG level [mg/dl, median (IQR)]	938.0 (593.0 – 1359.0)	942.0 (615.0 – 1367.0)	597.0 (250.0 – 1017.0)	< 0.001	812.0 (414.0 – 1277.0)	1012.0 (644.0 – 1430.0)	597.0 (250.0 – 1017.0)	< 0.001
28-day mortality [*n* (%)]	107 (6.2)	89 (5.5)	18 (19.6)	< 0.001	11 (6.0)	3 (3.3)	8 (8.7)	0.214
SOFA score [median (IQR)]	6.0 (4.0 – 9.0)	6.0 (4.0 – 9.0)	7.0 (4.8 – 10.0)	0.027	6.0 (5.0 – 10.0)	6.0 (5.0 – 10.0)	7.0 (4.8 – 10.0)	0.735
MV [*n* (%))	693 (40.3)	656 (40.3)	37 (40.2)	> 0.999	76 (41.3)	39 (42.4)	37 (40.2)	0.881
CRRT [*n* (%)]	133 (7.7)	119 (7.3)	14 (15.2)	0.010	26 (14.1)	12 (13.0)	14 (15.2)	0.832
Male [*n* (%)]	958 (55.7)	913 (56.1)	45 (48.9)	0.215	88 (47.8)	43 (46.7)	45 (48.9)	0.883
Age [years, median (IQR)]	63.1 (52.8 – 71.8)	63.3 (53.1 – 72.0)	58.9 (45.5 – 68.2)	0.004	58.0 (44.2 – 66.2)	56.8 (42.5 – 65.3)	58.9 (45.5 – 68.2)	0.303
Platelet [$\times 10^9$/L, median (IQR)]	83.0 (34.0 – 143.0)	85.5 (38.0 – 144.0)	24.0 (8.0 – 77.0)	< 0.001	30.0 (9.8 – 82.2)	31.0 (17.0 – 94.5)	24.0 (8.0 – 77.0)	0.131
Liver disease [*n* (%)]	712 (41.4)	672 (41.3)	40 (43.5)	0.758	85 (46.2)	45 (48.9)	40 (43.5)	0.554
Renal failure [*n* (%)]	778 (45.2)	739 (45.4)	39 (42.4)	0.649	77 (41.8)	38 (41.3)	39 (42.4)	> 0.999

Data were presented as *n* (%) for categorical variables and median (IQR) for continuous variables. *CRRT* continuous renal replacement therapy, *IgG* immunoglobulin G, *IQR* interquartile range*,* *IVIg* intravenous immunoglobulin, *MIMIC-IV* Medical Information Mart for Intensive Care IV, *MV* mechanical ventilation, *PSM* propensity score matching, *SOFA* sequential organ failure assessment

Table S10 Association of IVIg treatment with 28-day mortality after PSM in low IgG patients in the Qilu and MIMIC-IV cohorts [*n* (%)]

Variables	Qilu cohort[a]					MIMIC-IV cohort[b]				
	Total (*n* = 64)	Survivors (*n* = 28)	Non-survivors (*n* = 36)	*OR* (95% CI)	*P*-value	Total (*n* = 106)	Survivors (*n* = 100)	Non-survivors (*n* = 6)	*OR* (95% CI)	*P*-value
**Non-IVIg**	32 (50.0)	8 (28.6)	24 (66.7)	Ref.		53 (50.0)	49 (49.0)	4 (66.7)	Ref.	
**IVIg**	32 (50.0)	20 (71.4)	12 (33.3)	0.21 (0.07 – 0.59)	0.003	53 (50.0)	51 (51.0)	2 (33.3)	0.29 (0.10 – 0.75)	0.009

[a] In the Qilu cohort, low IgG was defined as serum IgG < 656 mg/dl. Model = IgG + age + sex + APACHE II + SOFA + MV + CRRT + comorbidities. [b] In the MIMIC cohort, low IgG was defined as serum IgG < 694 mg/dl. Model = IgG + age + sex + race + SOFA + MV + CRRT + comorbidities. Data were presented as *n* (%). *APACHE* II acute physiology and chronic health evaluation II, *CI* confidence interval, *CRRT* continuous renal replacement therapy, *IgG* immunoglobulin G, *IVIg* intravenous immunoglobulin, *MIMIC-IV* Medical Information Mart for Intensive Care IV, *MV* mechanical ventilation, *OR* odds ratio, *PSM* propensity score matching, *SOFA* sequential organ failure assessment
